# Detection of West Nile Virus, Usutu Virus and Insect-Specific Bunyaviruses in *Culex* spp. Mosquitoes, Greece, 2024

**DOI:** 10.3390/v17111414

**Published:** 2025-10-23

**Authors:** Katerina Tsioka, Konstantina Stoikou, Vasilis Antalis, Elissavet Charizani, Styliani Pappa, Sandra Gewehr, Stella Kalaitzopoulou, Spiros Mourelatos, Anna Papa

**Affiliations:** 1 Medical School, Aristotle University of Thessaloniki, 54124 Thessaloniki, Greece; aik.tsioka@gmail.com (K.T.); klstoikou@hotmail.com (K.S.); s_pappa@hotmail.com (S.P.); 2 Ecodevelopment SA, 57010 Thessaloniki, Greece; antalis@ecodev.gr (V.A.); gewehr@ecodev.gr (S.G.); skal@ecodev.gr (S.K.); mourelatos@ecodev.gr (S.M.)

**Keywords:** West Nile virus, mosquitoes, Usutu virus, insect-specific viruses, Greece

## Abstract

Greece is one of the countries in Europe most affected by West Nile virus (WNV), and since 2010, when the virus caused a large outbreak with 197 human neuroinvasive cases, outbreaks occur almost every year. Mosquito surveillance is an indirect sign of virus circulation; therefore, the purpose of the study was the molecular detection of WNV in 45,988 *C. pipiens* s.l. mosquitoes collected during 2024 in four Regions of Greece and the genetic characterization of the virus strains. WNV was detected in 41 of 1316 (3.12%) *Culex* spp. mosquito pools. Next-generation sequencing was applied to the WNV-positive samples that had a high viral load. All WNV sequences belong to Cluster B of the sub-lineage Europe WNV-2A presenting a temporal clustering. The WNV infection rates varied highly across the Regions, regional units and months, being higher in Thessaly and Central Macedonia Regions, especially in July and September. All mosquito pools were also tested for Usutu virus (USUV), and one pool was found positive, with sequence clustering into the EU-2 lineage. A subset of mosquitoes (737 pools) was tested for additional viruses, and bunya-like viruses were detected in 6 pools with sequences clustering into four distinct subclades. The prompt detection of pathogenic viruses is helpful for the design of control measures, while the detection of insect-specific viruses provides insights into viral diversity and evolution.

## 1. Introduction

Mosquitoes of the *Culex* genus are vectors of several viral pathogens, including West Nile virus (WNV) and Usutu virus (USUV). Both viruses belong to the genus Orthoflavivirus in the *Flaviviridae* family [1] and circulate in nature in an enzootic cycle between mosquitoes (mainly of the *Culex* species) as vectors and birds as amplifying reservoir hosts, while humans and other mammalian species are dead-end hosts due to low level of viremia [2,3]. Several avian species are susceptible to WNV and USUV; USUV, in particular, causes mass mortality in birds, mainly blackbirds in Europe [4,5,6].

Human WNV and USUV infections are usually asymptomatic or present as mild febrile illness, while less than 1% of the WNV infections present as neuroinvasive disease (WNND), mainly encephalitis, meningitis or flaccid paralysis, with a fatality rate of approximately 15% [7]. Neuroinvasiveness is seen less often in USUV infections; however, an increasing number of neurological cases are being reported, mainly in immunocompromised patients, with few of them with a fatal outcome [8,9,10,11,12]. It is of interest that several USUV infections have been detected in asymptomatic blood donors who tested WNV-positive in the nucleic acid amplification tests due to cross-reactivity between these two viruses [8,13]. 

Both WNV and USUV circulate in Europe; however, there is a big difference in the incidence and severity of the disease they cause. While WNV causes human outbreaks in southern and central Europe, the number of reported USUV infections is much lower, either because the circulation of the virus is limited, or due to the mild symptomatology of the disease, which remains unrecognized, or even due to misdiagnosis as WNV infections because of the cross-reactivity in serology. A recent study showed that in contrast to WNV, USUV cannot infect motor neurons in healthy individuals due to its restriction by the antiviral immune response, which could explain the differences in the clinical impact of these two viruses [14]. In addition, a study based on the human blood–brain barrier (BBB) model showed that USUV can replicate efficiently in BBB cells and promote immune activation, but the level of neuroinflammation is lower than that of WNV [15].

Since the geographical distribution of WNV and USUV in Europe is overlapping, there is a potential for co-infections in mosquitoes, which can influence the virus transmission. In vitro studies showed that co-infections might lead to a decreased growth of USUV in mosquitoes and of both viruses in vertebrate hosts [16]. A reduction in USUV transmission was seen in mosquitoes exposed simultaneously to both viruses compared to mosquitoes exposed only to USUV, while the infection and transmission of WNV was unaffected; in contrast, WNV transmission was significantly reduced when mosquitoes were pre-infected with USUV [17].

Both viruses are characterized by great genetic variability. Globally, nine WNV lineages have been identified (WNV-1 to WNV-9); however, most human infections are associated with WNV-1 and WNV-2. The predominant WNV lineage in recent years in Europe is WNV-2, which is divided into two sub-lineages: 2A and 2B. Specifically, sub-lineage WNV-2A emerged in Hungary around 2003–2004, and has diverged into Cluster A, which emerged in July 2006 and spread to northwest and western Europe, and Cluster B, which emerged in 2007 and spread to southern Europe, including Greece [18]. Similarly, the known USUV sequences cluster into eight lineages, Europe (EU)1 to EU5 and Africa (AF)1 to AF3. All lineages, except AF1, are present in Europe, suggesting that several virus introductions have occurred [6].

Greece is one of the most endemic countries for WNV in Europe. Specifically in 2024, it was the second-most affected country in EU/EEA after Italy [19]. The virus emerged in the country in the summer of 2010, in Central Macedonia Region, and caused a large outbreak with 197 WNND cases [20]. Since then, outbreaks occur annually, except 2016 and 2017, and up to the end of 2024, 1469 WNND cases (18% fatal) have been reported to the National Public Health Organization [21]. Molecular screening of mosquitoes for WNV infection is often used as an indirect sign of the virus circulation and as an early warning system, which facilitates the design of mosquito control measures. Previous studies in Greece showed that the WNV infection rate (IR) of *Culex* mosquitoes ranges from 0% to 17%, differing in years, months, regions, and regional units [22,23,24,25,26,27,28,29]. Regarding USUV, there is one report of virus detection in *Culex* mosquitoes in Greece [30], while in 2024, the virus was isolated from an asymptomatic blood donor (article in preparation). Therefore, the aim of the present study was the detection of WNV and USUV in *Culex pipiens* s.l. (hereafter *C. pipiens*) mosquitoes collected during 2024 and the genetic characterization of the virus strains. In addition, a generic RT-PCR, initially designed to detect phleboviruses, was applied in a subset of the mosquito collection.

## 2. Materials and Methods

### 2.1. Study Area

Mosquito collection was conducted in 250 sites in both urban and rural areas in four Regions of Greece. Specifically, mosquitoes were collected from 116 sites in Central Macedonia, 13 sites in West Macedonia, 75 sites in Thessaly, and 46 sites in West Greece. The regional units (RUs) tested per Region are shown in Table 1.

The collection sites were selected based on ecosystems favorable for mosquito breeding, such as proximity to rice fields, wetlands, and other water bodies, or in urban parks; areas where human cases had been reported in previous years were also included.

### 2.2. Mosquito Collection and Species Identification

Mosquitoes were collected by the Ecodevelopment mosquito control company using CO_2_-baited light traps with a constant CO_2_ outflow of 0.5 L/min (known as “Ecodev traps”). The traps were placed at the ground level in the morning and retrieved after approximately 24 h. The collection was conducted over a six-month period, spanning from May to October 2024, corresponding to the *Culex* spp. mosquito activity and abundance [31]. Each location was visited every two weeks, while additional traps were set at the locations where human or equine cases were recorded.

The mosquitoes were identified using a combination of morphological identification keys, such as arrangement of scales on the wings, the shape of the antennae, and the structure of the genitalia [32,33]. Female *C. pipiens* mosquitoes were transported to the laboratory on dry ice and stored at −80 °C until processing.

### 2.3. RNA Extraction

The mosquitoes were grouped into pools (up to 50 mosquitoes per pool), based on collection site and sampling date. The specimens were then rinsed with distilled water to remove any surface contaminants and homogenized in phosphate-buffered saline using glass beads (diameter 150–212 μm) in a TissueLyser II cell disrupter (Qiagen, Hilden, Germany) at 30 Hz for 3 min to break down the mosquito tissues and release the intracellular content. The total RNA was extracted from 200 μL supernatant of each homogenized pool using the IndiSpin Pathogen Kit (Qiagen, Hilden, Germany) and eluted in 50 µL RNase-free water following the manufacturer’s instructions.

### 2.4. Molecular Detection of Viruses

All mosquito pools were tested for qualitative detection of WNV and USUV. For WNV, a commercial real-time RT-PCR kit (West Nile Virus Real-TM, Sacace Biotechnologies Srl, Como, Italy) was applied, while for USUV, a conventional real-time RT-PCR with degenerate primer sets targeting conserved regions of the viral L genome segment was used [34]. The USUV-positive samples were further tested by a semi-nested RT-PCR using one forward primer (FU1) and two reverse primers (CFD3 and CFD2), which amplify a 265bp fragment of NS5 gene of flaviviruses [35]. The WNV and USUV infection rates (IRs) were estimated by dividing the number of positive pools by the total number of tested pools.

The mosquitoes of Central Macedonia were further tested using a conventional RT-nested PCR with degenerate primer sets, which was initially designed to target a region of the viral L genome segment of phleboviruses that encodes the RNA-dependent RNA polymerase (RdRp) [36].

### 2.5. Sanger Sequencing and Phylogenetic Analysis of USUV and Bunya-like Viruses

The PCR products of the conventional RT-PCRs were Sanger-sequenced in a 3130 ABI Genetic Analyzer (Applied Biosystems, Foster City, CA, USA). The nucleotide sequences were analyzed using the National Center for Biotechnology Information (NCBI) Basic Local Alignment Sequence Tool (BLAST) search engine (https://blast.ncbi.nlm.nih.gov/, accessed on 9 July 2025) to identify the best match.

USUV and bunya-like virus sequences were aligned with respective ones retrieved from the GenBank Database using Clustal W2, while evolutionary analyses and construction of the maximum likelihood phylogenetic trees based on the best-fitted nucleotide substitution model were conducted using MEGA version 12 [37].

### 2.6. Next-Generation Sequencing and Phylogenetic Analysis of West Nile Virus

The WNV-positive mosquito pools with a cycle threshold (Ct) of less than 30 in the WNV real-time RT-PCR were further processed using a PCR-based next-generation sequencing (NGS) protocol [38]. The libraries were prepared using the Ion 510 & Ion 520 & Ion 530 for Ion Chef kit for 400 base-reads and quantified using the Ion Library TaqMan Quantitation kit. Then, they were normalized to a final concentration of 35pM and sequenced on an Ion Torrent S5 platform using an Ion 520 semiconductor sequencing chip following the manufacturer’s instructions. All reagents were obtained from Life Technologies Corporation (Grand Island, NY, USA).

Raw reads were processed through the Torrent Suite Software version 5.18.1 for quality control, and the sequences were aligned using the sequence of Nea Santa-Greece-2010 strain (GenBank Accession number HQ537483) as reference. Assembly and annotation of the WNV whole-genome sequences were carried out using Geneious version 7.1.3. Reads were mapped to the refence sequence (HQ538473), with medium sensitivity/Fast, Fine-Tuning option Iterate up to 5 times, while the consensus sequences were generated with a minimum coverage of 20×, while all other parameters were used as the default values. The consensus sequences were aligned with WNV lineage 2 sequences obtained from the GenBank Database, and a maximum likelihood phylogenetic tree was generated using the best model in MEGA version 12 software [37].

### 2.7. Data Visualization

**Figure 1:**
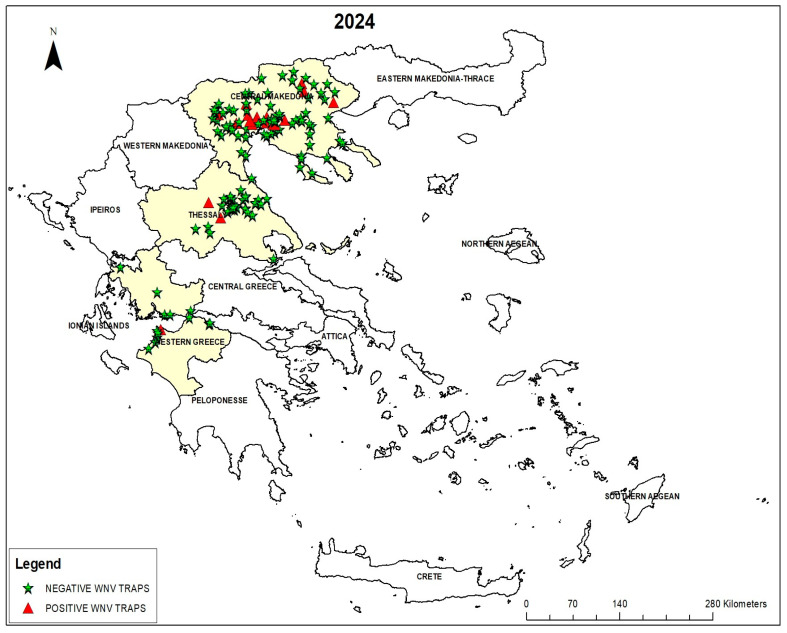
Map of Greece with the sites where WNV-positive and -negative *C. pipiens* mosquitoes were trapped in four Regions of Greece from May to October 2024.

The locations of the mosquito traps were mapped using the ArcGIS Pro geographic information system (GIS) application, version 3.0 (Esri, Redlands, CA, USA). The sites where WNV-positive mosquito pools were collected are shown on the map in red color (Figure 1).

## 3. Results

A total of 45,988 *C. pipiens* mosquitoes were collected and grouped into 1316 pools. WNV was detected in 41 pools (IR 3.12%). Specifically, 26 positive pools were detected in Central Macedonia (3.53%), 10 in Thessaly (3.98%), and 5 in West Greece (1.74%), while all mosquitoes from West Macedonia were negative (Table 1, Figure 1). The IR varied highly across the Regions, Rus, and months. The first evidence of WNV circulation was on 15 May 2024, in Thessaly Region. Highest IRs were recorded in July (5.37%) and September (4.47%). In four RUs (Larisa, Pieria, Ilia, and Achaia), the detection of WNV in mosquitoes preceded the onset of symptoms in human cases by 15–30 days. The location and date of collection of the positive pools are seen in Table 2. In one location in Pieria RU, WNV-positive mosquitoes were detected in three consecutive months (July, August, and September).

**Figure 2:**
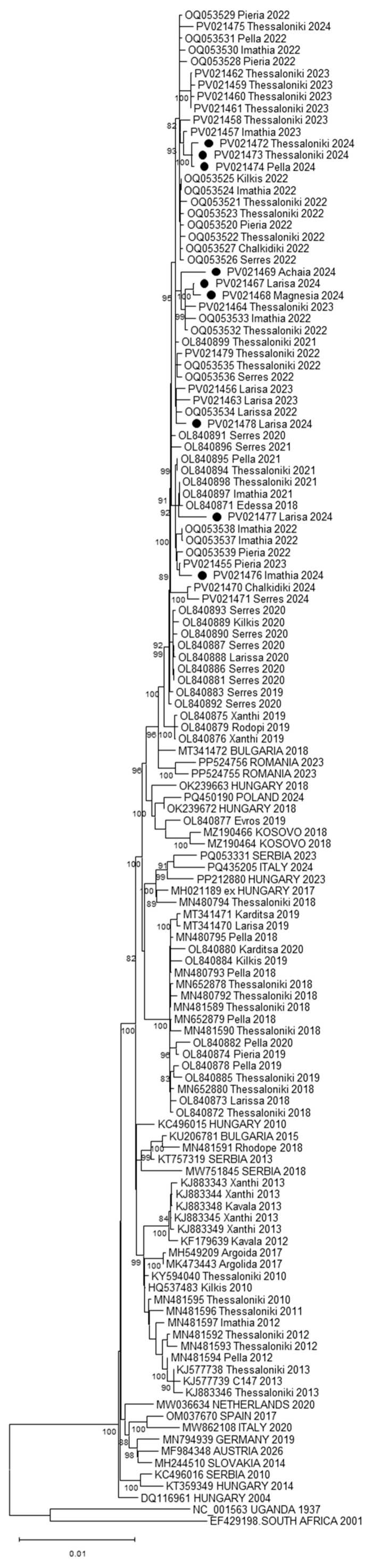
Maximum Likelihood phylogenetic tree based on whole-genome consensus sequences encoding the West Nile virus polyprotein (10,302 nucleotides). The percentage of replicate trees in which the associated taxa clustered together (100 replicates) is shown below the branches; only values > 75% are shown. The evolutionary rate differences among sites were modeled using a discrete Gamma distribution across 5 categories (+G, parameter = 1.2424), with 52.88% of sites deemed evolutionarily invariant (+I). The sequences of the present study are marked with a black circle. Sequences from Greece are shown as accession number, regional unit, and year of detection; sequences from other countries are shown in capital letters as accession number, country, and year of detection.

Whole-WNV genome sequences were taken by NGS from nine positive mosquito pools, which had a Ct value of less than 30 in the real-time RT-PCR. Phylogenetic analysis showed that all sequences of the current study belong to Cluster B (previously known as southeastern clade) of the sub-lineage Europe WNV-2A (Figure 2). A temporal clustering is seen among the Greek WNV strains; the sequences of 2024 cluster in a distinct subclade which contains sequences from Greece since 2018, differing by approximately 0.5% from sequences detected in previous years in the country.

**Figure 3:**
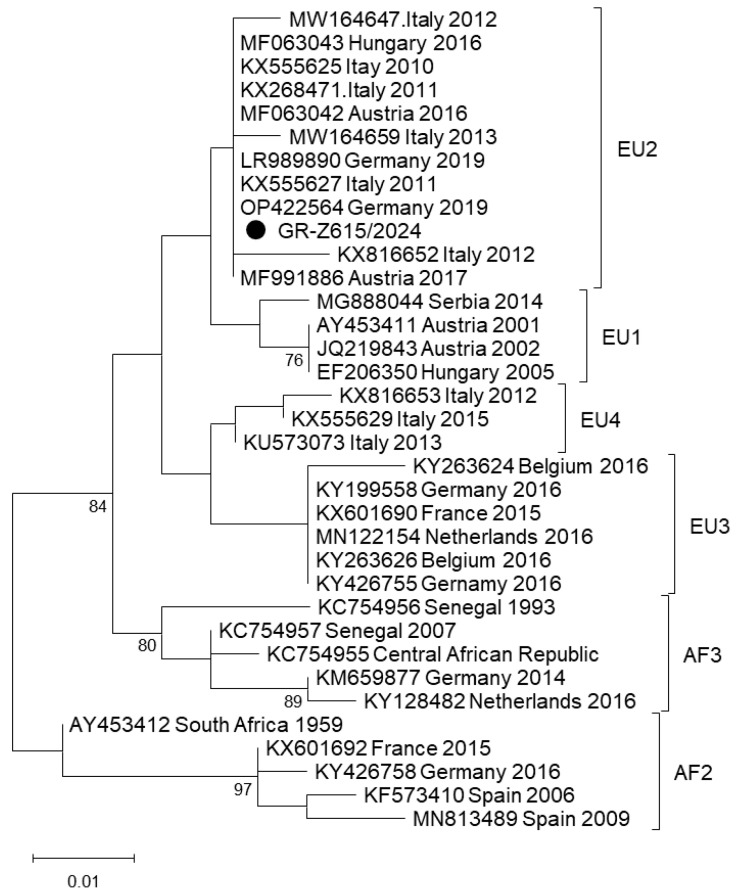
Maximum Likelihood phylogenetic tree based on a 212-bp fragment of USUV polyprotein gene. The tree was constructed using the Kimura-2 parameter model. The percentage of replicate trees in which the associated taxa clustered together (100 replicates) is shown below the branches; only values > 75% are shown. The sequence of the present study is marked with a black circle. Sequences are shown as accession number, country, and year of detection.

USUV was detected in one pool of mosquitoes (1/1316 pools, IR 0.07%), which were collected in September 2024 in Serres RU in Central Macedonia Region [IR for Central Macedonia 0.13% (1/737 pools)]. The result of the real-time RT-PCR was confirmed by sequencing of the product of the conventional semi-nested RT-PCR. The sequence presented 100% nucleotide identity with USUV sequences belonging to EU2 lineage (Figure 3).

**Figure 4:**
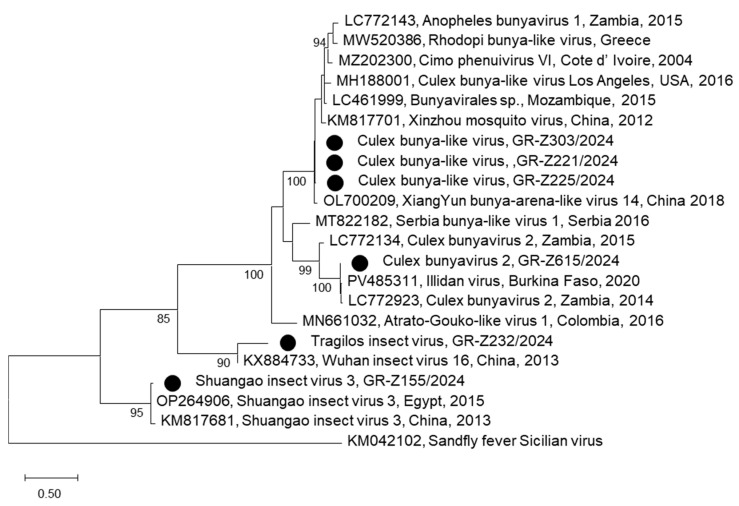
Maximum Likelihood phylogenetic tree based on 160 amino acid sequences of the viral-encoded RNA polymerase of bunyaviruses. The percentage of replicate trees in which the associated taxa clustered together (100 replicates) is shown below the branches; only values >75% are shown. The evolutionary rate differences among sites were modeled using a discrete Gamma distribution across 5 categories (+G, parameter = 1.2600), with 8.02% of sites deemed evolutionarily invariant (+I). The sequences of the present study are marked with a black circle. Sequences are shown as accession number, virus, country and year of detection; sequences from other countries are shown in capital letters as accession number, country, and year of detection.

The mosquitoes collected in Central Macedonia Region (737 pools) were also tested using a PCR which was originally designed to detect phleboviruses (genus phlebovirus, family *Phenuiviridae*). PCR products of the expected size were taken from six mosquito pools. BLAST analysis showed that the sequence of one pool shared 92% nucleotide identity with Shuangao insect virus 3, a second pool shared 87% identity with *Culex* bunyavirus 2, the sequences of three pools shared 83% identity with Xiang Yun bunya-arena-like virus 14, and the sequence of the sixth pool shared 73% identity with Wuhan insect virus 16. The sequences form four subclades, which are distinct from viruses of the phlebovirus genus. Specifically, GR-Z155/2024 clusters with Shuangao insect virus (unclassified virus in the *Peribunyaviridae* family); GR-Z221/2024, GR-Z225/2024, and GR-Z303/2024 cluster with Xiang Yun bunya-arena-like virus 14 (unclassified bunyavirus); GR-Z615/2024 clusters with *Culex* bunyavirus 2 (unclassified bunyavirus); and GR-Z232/2024 clusters with Wuhan insect virus 16 (unclassified RNA virus) (Figure 4). The Greek sequences were named according to the viruses of the subclade they are clustering, while a new name, Tragilos insect virus, was provisionally given to GR-232/2024, as it is distantly related to the only available virus in the subclade.

## 4. Discussion

Screening mosquitoes for WNV provides valuable information about spatial and temporal virus circulation in a region which is important for the design of prevention and control measures. The current study showed that 3.12% of the *C. pipiens* were WNV-positive, with the highest IRs seen in Thessaly and Central Macedonia Regions. Specifically, the IR was 3.98% in Thessaly, 3.53% in Central Macedonia, 1.74% in West Greece, and 0% in West Macedonia (Table 1). Although the geographic distribution of human cases in 2024 was almost countrywide, 70.5% (110/156) of WNND cases were reported from Thessaly, Central Macedonia, and West Greece, while no cases were reported in West Macedonia [21]. Based on a continuous phylogeographic model, it was shown that WNV-2A is attracted to areas with high crop and vegetation density, livestock cultivation and urbanization, as well as to wetlands, protected bird and habitat areas, and migratory bird flyways [18]. The largest plains with the most extensive agricultural production in Greece are in Thessaly and Central Macedonia, where the highest IRs were detected. Livestock farming is also highly concentrated in these two Regions and in West Greece. The same drivers are related also to increased transmission velocity of WNV-2A [18], which may explain the high number of clinical cases in these areas. Furthermore, major wetlands are present in Central Macedonia, while Thessaly experienced a catastrophic flood in September 2023 due to the storm Daniel, which actually did not affect the number of WNV cases in 2023 (it was already end of the WNV season), but might play a role in local ecosystem changes affecting the WNV circulation in 2024 [39,40].

The first detection of WNV in mosquitoes was in mid-May, earlier than in previous years (early to mid-June), suggesting a longer period of virus activity. The IRs in July and September were higher than in August when prolonged heat waves were observed in Greece. The hot weather during August might decrease the abundance of *C. pipiens* mosquitoes since extremely high temperatures reduce the abundance of mosquitoes [41].

Central Macedonia is the Region where WNV emerged in Greece in 2010; since then, it is the most entomologically studied area by our group. Investigations contacted during 2010 to 2024 in this area showed that the WNV IR of *Culex* spp. mosquitoes ranged from 0% to 9.6% (median 2.04%, Table 3) [22,23,25,31,42], and unpublished data}. The WNV IR in mosquitoes was 0% in 2014–2017, which coincides with the number of human WNND cases in this Region (Table 3). The discrepancy between the low IR and the highest number of human cases in 2010 could be explained by the absence of immunity in the naïve human and avian population prior to the emergence of the virus.

In a study conducted in twelve Regions of Greece during 2014–2016, WNV was detected in 1.17% (6/514) *C. pipiens* pools in 2014, 7.96% (9/113) pools in 2015 and 10.57% (22/208) pools in 2016 [24]. Sporadic studies in various areas in the country showed IRs ranging from 0% to 17% depending on the area, year, and number and size of the mosquito pools tested [26,27,28,29,44]. Variations in *C. pipiens*’ WNV positivity are also seen among European countries. As an example, WNV was detected in 12.2% (5/41) pools during 2018–2019 in Bulgaria [45], 5% (232/2337) in Emilia–Romagna and Lombardy regions (northern Italy) in 2018 [46], 2.31% (23/995) and 2.09% (20/956) in 2014 and 2015, respectively, in Serbia [47], while a study in Bucharest (Romania) showed that 21.7% (37/170) pools were positive in 2017, 17.3% (27/156) in 2018, 10.31% (23/223) in 2019, 2.88% (14/486) in 2020, 10% (21/210) in 2021, 7.77% (7/90) in 2022, and 8.06% (10/124) in 2023 [48]. Since IR depends on several parameters which differ between studies, comparison is not possible. However, it is seen that WNV mosquito screening is applied in several countries, especially the endemic ones, providing important information about the spatial and temporal circulation of the virus. In the present study, positive mosquito traps were found in one specific site for three consecutive months (July, August, and September), indicating that this site was highly affected, prompting for strengthening efforts to reduce the mosquito populations.

In four RUs (Larisa, Pieria, Ilia, and Achaia), the detection of WNV in mosquitoes preceded the human cases by 15–30 days, suggesting that sustained mosquito surveillance has the potential to serve as an early warning tool of viral activity allowing for timely intervention and application of prevention measures.

As in previous years, all WNV sequences of 2024 cluster into Cluster B of the sub-lineage WNV-2A. One exception was seen in 2018, when one sequence was found to cluster into the sub-lineage 2B [49]. Phylodymanic studies showed that specifically in 2018, three novel, independent introductions from Hungary and Bulgaria occurred in northern Greece [50]. At least 19 transmission events between Greece and other European countries occurred in the past decade, with Hungary, Serbia, and Romania being the countries with the most frequent events of virus transmission to Greece [18]. A limitation of the study was that the sequences were taken from pools of mosquitoes and not from individual mosquitoes, with a risk that more than one mosquito in the pool found to be positive. However, the pooling was performed based on the collection site and sampling date. Furthermore, the genetic distance between viruses of 2018–2024 is only 0.5% from those detected prior 2018.

Regarding USUV, the total IR was 0.07%; specifically, it was in Central Macedonia Region was 0.13% (1/737 pools) and 0% in West Macedonia, Thessaly, and West Greece. Similarly, in a previous study conducted in Greece during 2020–2023, USUV was detected only in Central Macedonia Region with IR of 1.03% (4/386 pools), while it was 0% in Thessaly (0/126 pools), suggesting a low-level virus circulation in the country [30]. Low USUV IRs in mosquitoes have been reported in several countries in southern and central Europe, mainly in *C. pipiens*, but also in other mosquito species, like *Aedes* and *Anopheles* [51,52,53,54,55,56,57]. The USUV-positive pool was detected in September. Similarly in Italy, USUV circulation was demonstrated in autumn (mid-October), while it was poorly detected in the summer [57]. By investigating the spatial spread in Europe, it was shown that Italy acted as main donor of USUV to neighboring countries [51]. The sequence taken from the Greek USUV-positive mosquito pool clusters with sequences of the EU2 lineage (Figure 3). Although the virus circulation in Greece is low, more studies in humans, mosquitoes, and birds are needed to gain a better insight into the USUV epidemiology in the country.

Co-infection with WNV and USUV in mosquitoes was not detected in this study. However, since both viruses are endemic in Europe, the competition between them during co-infections may reduce the vector competence of the USUV-infected mosquitoes for WNV, while the circulation of USUV in the WNV-free regions may prevent WNV transmission and spread [17]. Further field studies will show whether USUV can affect the epidemiology of WNV in Europe.

The rapidly increasing advances in genome sequencing technologies, combined with the availability of efficient bioinformatic tools, resulted in the identification of numerous novel viruses closely related to the viruses classified until recently in the family *Bunyaviridae*. This family included both pathogenic and insect-specific viruses (ISVs, viruses restricted to replicating only in insects). Therefore, in 2017, the International Committee on Taxonomy of Viruses (ICTV) promoted the family to order *Bunyavirales* [58], while in 2024, after repeated and substantial revisions, the order was promoted to class *Bunyaviricetes* [59]. The sequences of the present study cluster into four distinct subclades together with other bunyaviruses. Since the phylogeny was based on a small fragment of RdRp, we preferred the term “bunya-like viruses”; analysis of larger sequences will show their exact designation in virus taxonomy. Most probably, they are ISVs, which are not pathogenic for humans or animals; however, ISVs are important in understanding viral diversity and evolution, as well as their potential impact on vector competence for arboviruses [60].

## 5. Conclusions

A plethora of drivers (biological, ecological, and behavioral) play a role in the emergence and spread of mosquito-transmitted viral diseases, and WNV is a nice example for all these interactions [61]. The present study provided information about the spatial and temporal dynamic patterns of WNV in *C. pipiens* mosquitoes in four Regions of Greece in 2024 and showed that the highest WNV IRs were seen in two neighboring Regions, Thessaly and Central Macedonia, which are characterized by intense agricultural and pastoral activities, as well as presence of water bodies. Additionally, valuable insight into the molecular epidemiology of the disease was gained by enriching the WNV phylogeny with whole genome sequences from 2024. Regarding USUV, it was shown that the virus circulation in Greece is currently low, while the detection of insect-specific, bunya-like viruses prompts for further studies to understand better their distribution and evolution.

## Figures and Tables

**Table 1:** Detection of West Nile virus in *Culex pipiens* s.l. mosquitoes per month and regional unit in the Regions of Central Macedonia (CM), West Macedonia (WM), Thessaly (TH), and West Greece (WG), 2024. P: number of *C. pipiens* s.l. pools; M: number of *C. pipiens* s.l.

Regional Unit	May			June			July			August			September			October			Total		
	M	P	Pos P (%)	M	P	Pos P (%)	M	P	Pos P (%)	M	P	Pos P (%)	M	P	Pos P (%)	M	P	Pos P (%)	M	P	Pos P (%)
Imathia	237	9	0 (0)	572	17	0 (0)	830	22	1 (4.55)	602	16	1 (6.25)	880	17	0 (0)	0	0	0 (0)	3121	81	2 (2.47)
Thessaloniki	1383	43	0 (0)	1944	62	0 (0)	3162	97	7 (7.22)	2180	67	1 (1.49)	2564	69	4 (5.80)	0	0	0 (0)	11,233	338	12 (3.55)
Kilkis	120	5	0 (0)	301	10	0 (0)	357	13	0 (0)	194	9	1 (11.11)	240	11	0 (0)	0	0	0 (0)	1212	48	1 (2.08)
Pella	251	6	0 (0)	416	9	0 (0)	710	13	0 (0)	968	14	0 (0)	948	15	2 (13.33)	0	0	0 (0)	3293	57	2 (3.51)
Pieria	103	5	0 (0)	185	9	0 (0)	498	19	2 (10.53)	620	16	2 (12.50)	1160	19	1 (5.26)	0	0	0 (0)	2566	68	5 (7.35)
Serres	216	12	0 (0)	383	13	0 (0)	869	21	0 (0)	526	16	2 (12.50)	577	17	2 (11.76)	0	0	0 (0)	2571	79	4 (5.06)
Chalkidiki	202	9	0 (0)	315	11	0 (0)	539	16	0 (0)	582	14	0 (0)	717	16	0 (0)	0	0	0 (0)	2355	66	0 (0)
Subtotal CM	2512	89	0 (0)	4116	131	0 (0)	6965	201	10 (4.98)	5672	152	7 (4.61)	7086	164	9 (5.49)	0	0	0 (0)	26,351	737	26 (3.53)
Grevena	0	0	0 (0)	14	1	0 (0)	11	1	0 (0)	0	0	0 (0)	0	0	0 (0)	0	0	0 (0)	25	2	0 (0)
Kastoria	0	0	0 (0)	10	1	0 (0)	27	2	0 (0)	10	1	0 (0)	14	1	0 (0)	0	0	0 (0)	61	5	0 (0)
Kozani	50	2	0 (0)	53	1	0 (0)	53	3	0 (0)	50	3	0 (0)	70	4	0 (0)	0	0	0 (0)	276	13	0 (0)
Florina	0	0	0 (0)	27	2	0 (0)	281	7	0 (0)	335	7	0 (0)	123	5	0 (0)	0	0	0 (0)	766	21	0 (0)
Subtotal WM	50	2	0 (0)	104	5	0 (0)	372	13	0 (0)	395	11	0 (0)	207	10	0 (0)	0	0	0 (0)	1128	41	0 (0)
Karditsa	80	6	0 (0)	230	8	0 (0)	141	6	0 (0)	50	1	0 (0)	50	1	0 (0)	0	0	0 (0)	551	22	0 (0)
Larisa	1107	27	2 (7.41)	1046	24	0 (0)	3704	59	5 (8.47)	1951	41	0 (0)	1765	33	2 (6.06)	86	2	0 (0)	9659	186	9 (4.84)
Magnisia	124	3	0 (0)	45	2	0 (0)	87	7	0 (0)	129	5	0 (0)	330	12	1 (8.33)	154	7	0 (0)	869	36	1 (2.78)
Sporades	0	0	0 (0)	0	0	0 (0)	81	3	0 (0)	26	2	0 (0)	19	2	0 (0)	0	0	0 (0)	126	7	0 (0)
Subtotal TH	1311	36	2 (5.56)	1321	34	0 (0)	4013	75	5 (6.67)	2156	49	0 (0)	2164	48	3 (6.25)	240	9	0 (0)	11,205	251	10 (3.98)
Aitoloak/nia	371	13	0 (0)	339	13	0 (0)	145	11	0 (0)	78	10	0 (0)	221	18	0 (0)	125	9	0 (0)	1279	74	0 (0)
Axaia	446	16	0 (0)	482	14	0 (0)	1251	39	3 (7.69)	458	28	0 (0)	877	34	1 (2.94)	663	20	0 (0)	4177	151	4 (2.65)
Ileia	265	6	0 (0)	176	6	0 (0)	549	15	1 (6.67)	297	10	0 (0)	355	17	0 (0)	206	8	0 (0)	1848	62	1 (1.61)
Subtotal WG	1082	35	0 (0)	997	33	0 (0)	1945	65	4 (6.15)	833	48	0 (0)	1453	69	1 (1.45)	994	37	0 (0)	7304	287	5 (1.74)
**Total**	4955	162	2 (1.23)	6538	203	0 (0)	13,295	354	19 (5.37)	9056	260	7 (2.69)	10,910	291	13 (4.47)	1234	46	0 (0)	45,988	1316	41 (3.12)

**Table 2:** Location and collection date of the WNV-positive mosquito pools detected in the present study.

Pool ID	Collection Date	Regional Unit	Region
Z324	17 July 2024	Imathia	Central Macedonia
Z497	12 August 2024	Imathia	Central Macedonia
Z464	07 August 2024	Kilkis	Central Macedonia
Z618	02 September 2024	Pella	Central Macedonia
A305	16 September 2024	Pella	Central Macedonia
A168	15 July 2024	Pieria	Central Macedonia
A217	29 July 2024	Pieria *	Central Macedonia
A251	09 August 2024	Pieria *	Central Macedonia
A260	09 August 2024	Pieria	Central Macedonia
A311	09 September 2024	Pieria *	Central Macedonia
Z468-A	07 August 2024	Serres	Central Macedonia
Z468-B	07 August 2024	Serres	Central Macedonia
Z613	04 September 2024	Serres	Central Macedonia
Z643	04 September 2024	Serres	Central Macedonia
Z332	15 July 2024	Thessaloniki	Central Macedonia
Z355	15 July 2024	Thessaloniki	Central Macedonia
Z370	24 July 2024	Thessaloniki	Central Macedonia
Z373	24 July 2024	Thessaloniki	Central Macedonia
Z366	24 July 2024	Thessaloniki	Central Macedonia
Z417	31 July 2024	Thessaloniki	Central Macedonia
Z440	31 July 2024	Thessaloniki	Central Macedonia
A316	28 August 2024	Thessaloniki	Central Macedonia
Z627	04 September 2024	Thessaloniki	Central Macedonia
Z639	04 September 2024	Thessaloniki	Central Macedonia
Z611	04 September 2024	Thessaloniki	Central Macedonia
Z668	11 September 2024	Thessaloniki	Central Macedonia
A11	15 May 2024	Larissa	Thessaly
A16	15 May 2024	Larissa	Thessaly
A119	03 July 2024	Larissa	Thessaly
A153	05 July 2024	Larissa	Thessaly
A181	17 July 2024	Larissa	Thessaly
Z380	24 July 2024	Larissa	Thessaly
A183	24 July 2024	Larissa	Thessaly
Z623	04 September 2024	Larissa	Thessaly
A315	04 September 2024	Larissa	Thessaly
A327	11 September 2024	Magnesia	Thessaly
Z252-A	01 July 2024	Achaia	West Greece
Z252-B	01 July 2024	Achaia	West Greece
Z252-C	01 July 2024	Achaia	West Greece
Z617	04 September 2024	Achaia	West Greece
A151	03 September 2024	Ilia	West Greece

* Entries with an asterisk are referred to one specific location where WNV positive pools were serially detected.

**Table 3:** Infection rates (IRs) of mosquito pools tested by our group in Central Macedonia (CM) Region, Greece, 2010–2023. The number of human WNND cases from Central Macedonia reported to the National Public Health Organization are also shown [43].

Year	Positive Pools/Pools Tested (IR)	Reference	Human WNND Cases in CM
2010	3/224 (1.34)	[22]	186
2011	2/53 (3.77)	[22]	23
2012	0/100 (0)		15
2013	9/295 (3.1)	[23]	13
2014	0/207 (0)		0
2015	0/438 (0)		0
2016	0/62 (0)		0
2017	0/55 (0)		0
2018	10/229 (4.4)	[25]	78
2019	5/346 (1.5)	[31]	37
2020	13/362 (3.6)	[31]	76
2021	37/391 (9.6)	[31]	31
2022	41/ 690 (5.9)	[42]	148
2023	19/736 (2.58)		55
2024	26/737 (3.53)	current study	60

## Data Availability

The data presented in this study is available on request from the corresponding author.
